# GeM-LR: Discovering predictive biomarkers for small datasets in vaccine studies

**DOI:** 10.1371/journal.pcbi.1012581

**Published:** 2024-11-14

**Authors:** Lin Lin, Rachel L. Spreng, Kelly E. Seaton, S. Moses Dennison, Lindsay C. Dahora, Daniel J. Schuster, Sheetal Sawant, Peter B. Gilbert, Youyi Fong, Neville Kisalu, Andrew J. Pollard, Georgia D. Tomaras, Jia Li

**Affiliations:** 1 Department of Biostatistics and Bioinformatics, Duke University, Durham, North Carolina, United States of America; 2 Center for Human Systems Immunology, Duke University, Durham, North Carolina, United States of America; 3 Duke Human Vaccine Institute, Duke University, Durham, North Carolina, United States of America; 4 Department of Surgery, Duke University, Durham, North Carolina, United States of America; 5 Department of Integrative Immunobiology, Duke University, Durham, North Carolina, United States of America; 6 Vaccine and Infectious Disease Division, Fred Hutchinson Cancer Center, Seattle, Washington, United States of America; 7 Center for Vaccine Innovation and Access, PATH, Washington, DC, United States of America; 8 Oxford Vaccine Group, Department of Paediatrics, University of Oxford, Oxford, United Kingdom; 9 NIHR Oxford Biomedical Research Centre, Oxford, United Kingdom; 10 Department of Molecular Genetics and Microbiology, Duke University, Durham, North Carolina, United States of America; 11 Department of Statistics, The Pennsylvania State University, Pennsylvania, United States of America; University of Southern California, UNITED STATES OF AMERICA

## Abstract

Despite significant progress in vaccine research, the level of protection provided by vaccination can vary significantly across individuals. As a result, understanding immunologic variation across individuals in response to vaccination is important for developing next-generation efficacious vaccines. Accurate outcome prediction and identification of predictive biomarkers would represent a significant step towards this goal. Moreover, in early phase vaccine clinical trials, small datasets are prevalent, raising the need and challenge of building a robust and explainable prediction model that can reveal heterogeneity in small datasets. We propose a new model named Generative Mixture of Logistic Regression (GeM-LR), which combines characteristics of both a generative and a discriminative model. In addition, we propose a set of model selection strategies to enhance the robustness and interpretability of the model. GeM-LR extends a linear classifier to a non-linear classifier without losing interpretability and empowers the notion of predictive clustering for characterizing data heterogeneity in connection with the outcome variable. We demonstrate the strengths and utility of GeM-LR by applying it to data from several studies. GeM-LR achieves better prediction results than other popular methods while providing interpretations at different levels.

## Author summary

Vaccines have proven to be a powerful tool in preventing infectious diseases, yet their effectiveness can vary significantly from person to person. This variability underscores the need for a better understanding of how individuals’ immune systems respond to vaccination, which is essential for achieving successful immunization across a broader population. In our study, we introduce a new model called the Generative Mixture of Logistic Regression (GeM-LR) to predict vaccine effectiveness in different individuals. This model is particularly beneficial when only small datasets are available, a common challenge in vaccine research, whereas many advanced machine learning methods require large training datasets. GeM-LR integrates mixture modeling and logistic regression to provide more accurate predictions while also offering insights into the factors that contribute to varying vaccine responses among individuals. We demonstrate that our model outperforms other standard methods in terms of accuracy and enhances the understanding of data for future research. Our innovative approach holds great promise for improving vaccine development. By revealing hidden patterns within small datasets, GeM-LR aims to make vaccine research more efficient and impactful.

## 1. Introduction

Many machine learning methods have been applied successfully to binary outcome prediction problems in the biomedical research area, such as disease gene prediction [1,2], binary biomedical image segmentation [3], and binary disease outcomes prediction [4–7]. Biomedical data often feature multi-dimensional, complex distributions, with heterogeneous sources of noise, necessitating advanced modeling methods. To achieve accurate outcome prediction, these advanced methods, usually of high complexity, must be trained using a large training set. When analyzing small datasets, the advantage in predictive performance of these state-of-the-art methods diminishes. Given the difficulties in interpreting complex models, they are not appealing to use without a significant gain in predictive performance.

Small datasets are prevalent in early phase vaccine clinical trials, where the sample size (i.e., the number of participants in a study) ranges from tens to a few hundred. Despite many advances in vaccine development, the variability in the level of protection provided by a vaccine across individuals remains substantial and is primarily attributed to diverse immune responses within a population [8–13]. Such inter-individual variations in immune responses to vaccinations impose great challenges in both designing more effective vaccines and deploying them to the public. As a result, it is critical to understand and identify such variations to accurately predict which individuals have responded to vaccines and which have not. Furthermore, the ability to identify predictive biomarkers, i.e., correlates of protection, while predicting vaccine efficacy can be a powerful tool to expedite and benchmark vaccine development. Machine learning methods capturing individual variations can potentially achieve more accurate predictions. However, those methods will typically result in increased model complexity that can suffer from overfitting with small datasets and render less interpretable models. Thus, there is a need for developing robust and reliable methods for small datasets capable of addressing heterogeneity in data.

We have developed a new class of *Generative Mixture of Logistic Regression* (GeM-LR) models to overcome the aforementioned challenges. GeM-LR is designed to capture the variation in the relationship between the features and the outcome when the input vector resides in different regions of the feature space. As a result, two types of data variations are specified by a GeM-LR. First, the feature space is partitioned into regions, which exhibit variation between the input vectors. Second, the region- or cluster-wise predictive models vary in terms of the mapping from the input to the predicted output. For example, different biomarkers may be selected for prediction. The essential concept is to first uncover data heterogeneity in the feature space by partitioning the data into more homogeneous subgroups (clusters). Here, “homogeneous” means that not only are the feature vectors similar, but also the relationship between the outcome variables to be predicted and the feature vector is linearly coherent. The heterogeneity, on the other hand, is preserved and reflected as between-cluster variations. Assuming that each subgroup is homogeneous, we use linear classification models within each cluster. In particular, we choose the widely used linear logistic regression with sparsity regularization, which performs competitively for smaller sample sizes and yields an easy-to-explain prediction function. Essentially, the clustering analysis, to be done by a generative model, allows us to characterize a complex outcome prediction function by a composite of much simpler functions, an effective way to balance model complexity and flexibility. Most crucially, this strategy empowers the notion of predictive clustering, which allows individuals grouped together to share similar predictive biomarkers associated with their outcome variables. The joint estimation of parameters in GeM-LR also facilitates information sharing across clusters.

We also developed model selection procedures which help us decide how many clusters are needed and identify features most useful for annotating a cluster and those most effective for predicting the outcome of a given study (potential predictive biomarkers). Thus, contrary to many other machine learning methods, GeM-LR offers more model interpretability and sheds light on data heterogeneity. The capability to extract different predictive biomarkers for different groups of individuals may provide insight into why some individuals do respond to vaccines while others do not. Our modeling approach will provide essential information for understanding individuals’ predictive immune response patterns so that vaccine candidates can be prioritized to elicit the biomarkers associated with favorable outcomes [14–16]. In addition, GeM-LR, by design, is computationally very efficient and easy to implement, and the number of tuning parameters is minimal.

We demonstrate the utility of GeM-LR by applying the model to data from three vaccine trials. We also compare GeM-LR with popular machine learning methods, including logistic regression with elastic net penalty [17, 18], cluster-then-predict (CP), K-nearest neighbor (KNN) classification [19], random forest (RF) [20] and shallow neural network (SNN). The results demonstrate that GeM-LR can achieve higher predictive performance while providing insights into the data heterogeneity. Hence, it is a highly useful approach for analyzing vaccine studies.

## 2. Methods

In this section, we introduce the method of *Generative Mixture of Logistic Regression* (GeM-LR) model. Since logistic regression is a generalized linear model, we regard GeM-LR as a special case of *Generative Mixture of Linear Models* (GMLM). We also describe other competing methods and the model evaluation strategies.

### 2.1. The generative mixture of logistic regression models

We propose identifying data heterogeneity by grouping individual data points into homogeneous subgroups (clusters). The traditional clustering analyses rely only on a notion of similarity in the feature space (or a low-dimensional projection of the space) but neglect the individuals’ outcome information. In other words, there is no attempt to achieve a similar dependency between the outcome and the input vector for individuals in the same cluster. Different from the traditional clustering analyses, our method integrates the optimization of both predictive cluster detection and the estimation of cluster-specific predictive functions for the outcome. The estimation of GeM-LR implies that the predictive clusters are generated under the supervision of class labels. In addition to favoring tight clusters in the feature space, GeM-LR also strives for homogeneity in the dependency relationship between the input and the output. The homogeneity within each cluster is reflected by a good fit of a logistic regression (LR) model that predicts the outcome based on the input vector. The within-cluster LR model is easy to explain and reveals the predictive biomarkers.

**Fig 1:**
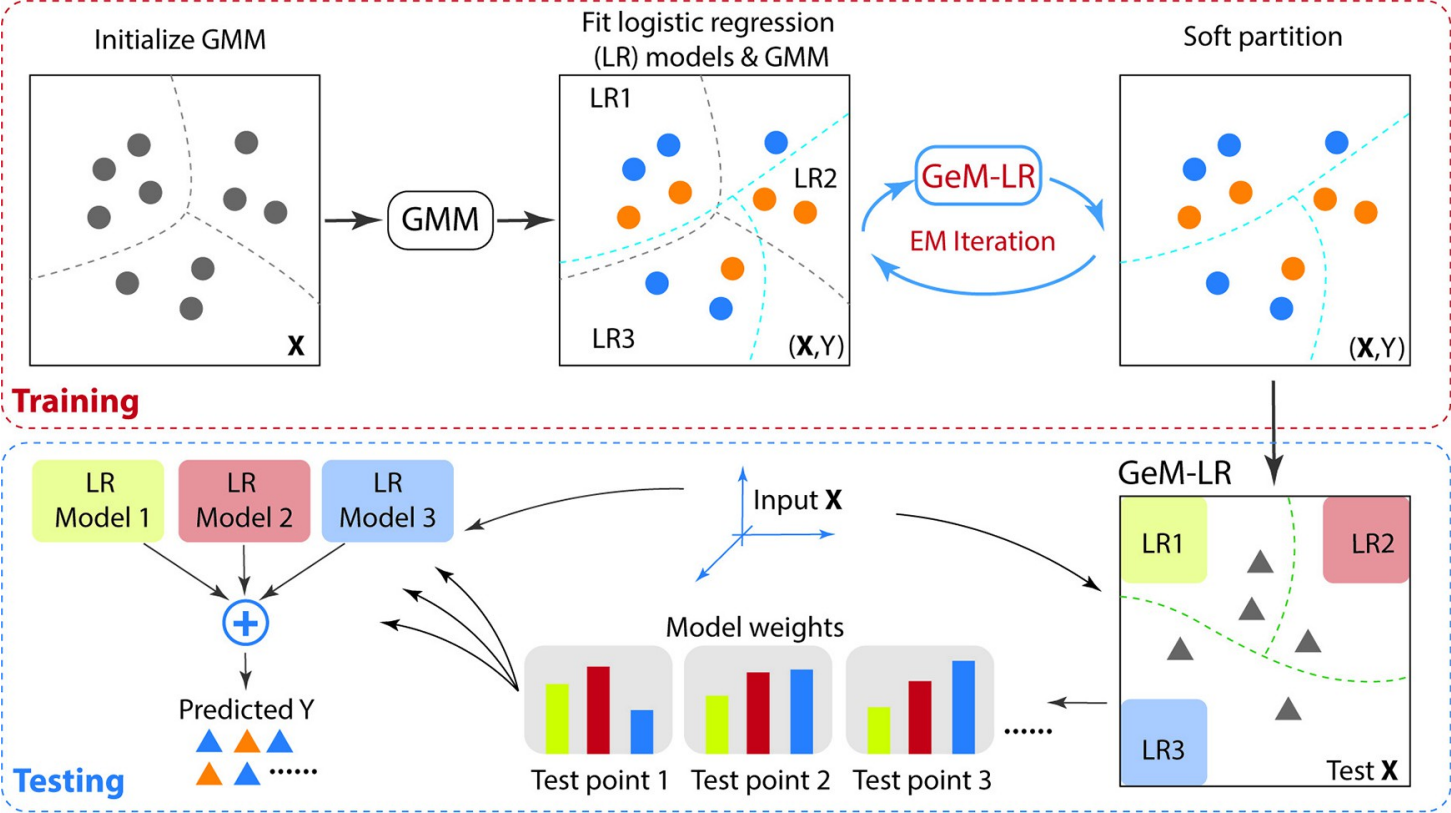
An illustration of the training and testing processes of GeM-LR. Top panel: The training process involves initializing the embedded GMM model through conventional GMM fitting without using *Y*. Then, GeM-LR is estimated by the Expectation-Maximization (EM) algorithm. In particular, the embedded GMM and LR models are jointly optimized by EM iterations. Bottom panel: The testing process begins with computing weights for each LR model based on the fitted GMM model for each test point. These weights are then used to calculate the weighted sum of the predicted class posterior probabilities of each LR model, which serves as the final prediction.

Fig 1 illustrates the idea of GeM-LR, which can be treated as a latent probabilistic graph model. To illustrate its structure, we first consider the conventional approach of clustering based on a Gaussian mixture model (GMM), which is popular in different fields [21–28]. During the model training stage, the GMM embedded within GeM-LR is initialized using conventional GMM fitting without considering the outcome variable. This initialization step corresponds to traditional clustering, where each mixture component in the GMM represents a cluster. Following this, GeM-LR is estimated using the Expectation-Maximization (EM) algorithm. Specifically, the embedded GMM and LR models within each cluster are jointly optimized through EM iterations. A noteworthy aspect of GeM-LR’s training pipeline is the simultaneous optimization of cluster formation and the fitting of cluster-wise models through iterative refinement. This novel process, grounded in robust mathematical principles, enhances the likelihood of identifying clusters with more effective predictive biomarkers. The testing process begins by computing weights for each LR model based on the fitted GMM model for each test data point. These weights are then used to calculate the weighted sum of the predicted class posterior probabilities from each LR model, which constitutes the final prediction.

Now we present the mathematical formulation of GeM-LR. Let the random vector **X**∈ℛ^*p*^ be the *p*-dimensional input feature vector and let **x** be a realization of **X**. The output is denoted by Y∈Y. In this paper, we focus on binary classification, and thus Y={0,1}. A GMM with *C* components has the density function: $P(X)=\sum_{c=1}^{C}\pi_{c}N(X|\mu_{c},\Sigma_{c})$, where *π*_*c*_ is the mixture component prior probability with $\sum_{c=1}^{C}\pi_{c}=1$ and *N*(.|***μ***_*c*_,**Σ**_*c*_) is the multivariate Gaussian density parameterized by ***μ***_*c*_ and **Σ**_*c*_,***μ***_*c*_ being the *p*-dimensional mean vector and **Σ**_*c*_ the *p*×*p* covariance matrix. GMM is typically used to perform probabilistic clustering in the unsupervised setting, where the similarity between the data points is measured purely based on the proximity of **x** quantified by *N*(.|***μ***_*c*_,**Σ**_*c*_), *c* = 1,…,*C*. To perform clustering, we assume a latent component/cluster label *Z*,*Z*∈{1,2,…,*C*}. An instance of *Z* is denoted by *z*. The marginal probability mass function for *Z* is given by *P*(*Z* = *c*) = *π*_*c*_,*c* = 1,…,*C*. GMM assumes that given *Z* = *c*,**X** follows a Gaussian distribution with mean ***μ***_*c*_ and covariance **Σ**_*c*_. We then assign a data point **x** to the cluster with the maximum posterior: $P(Z=c|X=x)\propto \pi_{c}N(x|\mu_{c},\Sigma_{c})$.

To extend GMM to GeM-LR, we propose to integrate a LR model within each cluster: $P(Y=1|X=x,Z=c)=\frac{\exp\left(\beta_{c}^{T}x+\beta_{c,0}\right)}{1+\exp\left(\beta_{c}^{T}x+\beta_{c,0}\right)}$, where ***β***_*c*_ = (*β*_*c*,1_,…,*β*_*c*,*p*_)^*T*^ are the linear coefficients in the *c*th linear model (i.e., *c*th cluster) and *β*_*c*,0_ is the intercept term. The cluster-specific logistic regression can identify predictive biomarkers within each cluster. Importantly, the cluster-specific regression is not conducted using only data points within that cluster. Instead, all the data points are involved in the estimation of any cluster-specific regression, while each point is weighted by how likely it belongs to that cluster. We can regard this weighted regression as a principled way of leveraging information from other clusters. As a result, the regression models can be more robustly estimated for small clusters. Furthermore, the determination of the cluster indicator *Z* is based on both the outcome variable and the feature vector. Thus, the predictive clusters are formed under the guidance of the class labels instead of via unsupervised learning. The detailed formulation of the GeM-LR is provided in S1 Appendix.

In summary, our GeM-LR model is a latent probabilistic graph model where the latent component *Z* is assumed with a discrete distribution; given *Z*, the input vector **X** follows a Gaussian distribution; and finally, given both *Z* and **X**,*Y* is modeled by a linear logistic regression with parameters depending on *Z*. If we consider the marginal distribution of **X**, we indeed end up with a GMM, which we call an embedded GMM in GeM-LR. Since GMM is generative while LR is discriminative, so we call our model Generative Mixture of Logistic Regression (GeM-LR) to distinguish from a Mixture of Linear Models (MLM), which often refers to a type of discriminative model. Although MLM has also been used as a short name for Generative Mixture of Linear Models (GMLM) in the literature [29]. In previously published terminology [29], both linear regression and linear logistic regression for classification are called linear models.

#### Model training and testing

To estimate GeM-LR, we use the EM algorithm with regularization where the latent state *Z* is regarded as missing data. Regularization is imposed on the linear logistic regression model. In particular, since the immune features in vaccine studies are typically highly correlated, we choose elastic net [18] for regularization and feature selection. Elastic net encourages grouping effects where a group of highly correlated features tends to be in or out of the model together. The training and testing processes for GeM-LR are illustrated in Fig 1. Details on the estimation algorithm for GeM-LR are provided in the S1 Appendix. During testing, we compute the predicted probability for class 1 given instance $X:P(Y=1|X)=\sum_{c=1}^{C}P(Z=c|X)P(Y=1|X,Z=c)$, which is obtained by taking the weighted sum of the posterior probabilities *P*(*Y* = 1|**X**,*Z* = *c*), with the weights corresponding to the posterior probabilities of the clusters *P*(*Z* = *c*|**X**).

#### Operational considerations

Despite the interaction between the estimation of the GMM and the estimation of the LR models, to classify a test data point, GeM-LR operating like a soft-partition version of the cluster-then-predict method (see Fig 1). First, the GMM is used to obtain posterior probabilities *P*(*Z* = *c*|**X**), *c* = 1.…,*C*, which act as weights on the predicted probabilities *P*(*Y* = 1) by the LR models. Since LR models offer good explainability, the result of GeM-LR can be interpreted as a weighted decision based on the LR models. We can also adopt hard partition during testing by setting *Z* = c*, the value that yields the maximum posterior. This scheme will make interpretation even easier since we pick only one LR model to predict the outcome.

Another flexibility provided by the GeM-LR model is that the input **X** modeled in the GMM can be different from **X** used in the LR models. Specifically, we may want to generate the partition of subjects using features different from those used to predict *Y* in the LR models. The motivation for this practice is multifold. For high dimensional data, although the LR models can use regularization to reduce dimension, the GMM may lack robustness against high dimensions. Therefore, we may choose to model only a subset of features in **X** by the GMM. In addition, domain experts may have prior knowledge about subpopulations of subjects and may want to partition them according to certain features, some or all of which can be meta-data not included in the input features of the LR models. This variation in GeM-LR causes negligible changes in the training and testing methods.

#### Regularization on small clusters

Several approaches are used to address the issue of small clusters, where the number of data points in a cluster is smaller than the dimensionality of the feature space used for clustering. The estimation of the component-wise covariance matrix in the GMM is mostly affected by a small data size. We have implemented a variety of options to regularize the covariance matrix, as is done in Mclust [30]. For instance, the covariance matrices across components can be assumed identical or they can share some characteristics. Each covariance matrix is decomposed such that the orientation, shape, and volume of the Gaussian component are separated. Then a user can choose whether to require all or part of these characteristics to be common across the components. The covariance matrix can also be set diagonal. Furthermore, we can estimate the covariance matrix using shrinkage towards a diagonal matrix with an adjustable level of shrinkage. The level of shrinkage is between (0,1). A larger value indicates more shrinkage towards diagonal covariance.

Furthermore, the GeM-LR model does not require that the features modeled by GMM are the same as the features used in the cluster-wise logistic regression model. Hence, we can use a higher dimensional feature vector to generate the logistic regression model but keep the dimension of the GMM low. We have explained previously how to select features to include in the GMM. For a tiny cluster impossible to fit an elastic net model, we assume the simplest classification model–predicting class posteriors by the empirical class frequencies in that cluster.

#### Determine the number of clusters

Given that the interest of modeling is to achieve accurate predictions, we choose as the optimal number of clusters the value that achieves the highest cross-validation predictive performance as described below in Section 2.3. There are various approaches to determining the optimal number of clusters. In this paper, we focus on predictive performance, given that our training data are labeled. In contrast, the number of clusters may also be selected in unsupervised settings, for instance, based on stability measures of clustering results [31].

#### Interpretation

Without loss of generality, we assume that GeM-LR is fitted on the full set of features. We propose two model selection procedures to interpret GeM-LR by (1) annotating the identified clusters and (2) selecting potential biomarkers of the outcome for each cluster. Since the clusters generated by GeM-LR are created in a supervised manner, interpreting the importance or usefulness of the features requires considering their roles at two stages. In the first stage, features are used to separate the clusters, while in the second stage, they help predict the class/outcome. As a result, the features selected by LR within clusters are not the only factors driving class prediction. For example, a feature crucial for cluster separation may not be selected by any LR models, yet it remains essential for distinguishing between clusters.

To annotate the clusters, we propose to identify the cluster-specific discriminative features. More specifically, we aim to prioritize the features in their ability to discriminate each cluster from the rest [32]. Then the understanding of a given cluster will be based on one or multiple features that have the most discriminative ability. For each cluster *c*,*c* = 1,…,*C*, we view this task of feature selection as a classification problem. We wish to identify a set of features that can discriminate cluster *c* with the highest accuracy. To measure the “accuracy”, we first compute true- and false-positive discriminative measures. Assuming a specific observation actually arises from cluster *c*, i.e., **x**~*N*_*c*_≡*N*(.|***μ***_*c*_,**Σ**_*c*_), the true positive to cluster *c* can be represented by

$$\tau_{c+}(h)=\frac{E[\pi_{c}N_{c}\left(x(h)\right)|x(h)∼N_{c}]}{E[P(x(h))|x(h)∼N_{c}]},$$

where *h*⊆{1:*p*} denotes a set of features. The idea is that the optimal set of features should make the cluster *c* well separated from the other clusters so that there is no overlap between *N*_*c*_ with *N*_*s*_, for *s*≠*c*. As a result, *τ*_*c*+_(*h*) will be close to 1. On the other hand, if $x∼P_{-c}=\frac{1}{\left(1-\pi_{c}\right)}\sum_{s=1:C,s\neq c}\pi_{s}N_{s}$, i.e., **x** does not arise from cluster *c*, the false-positive can be defined as

$$\tau_{c-}(h)=\frac{E[\pi_{c}N_{c}(x(h))|x(h)∼P_{-c}]}{E[P(x(h))|x(h)∼P_{-c}]}.$$

We would like to select a set of features that make *τ*_*c*−_(*h*) close to 0.

Thus, to combine both *τ*_*c*+_(*h*) and *τ*_*c*−_(*h*), we define the *aggregate discriminative accuracy measure*: $A_{c}(h)=\pi_{c}\tau_{c+}(h)+(1-\pi_{c})(1-\tau_{c-}(h))$ for each cluster *c* = 1:*C* and a given set of features *h*.*A*_*c*_(*h*) is the sum of true-positive (*τ*_*c*+_(*h*)) and true-negative (1- *τ*_*c*−_(*h*)) rates weighted by the corresponding cluster sizes. *A*_*c*_(*h*)∈[0,1] is on the absolute probability scale, so differences across different subsets *h* can be easily interpreted. A larger the value of *A*_*c*_(*h*) indicates that the subset *h* of features is discriminative for cluster *c*. Therefore, we can rely on the features in subset *h* to understand and annotate cluster *c*. More importantly, for GMM, both *τ*_*c*+_(*h*) and *τ*_*c*−_(*h*) have close form expressions, and thus *A*_*c*_(*h*) is easy to compute.

Given the fitted GMM on the full set of *p* features, we can directly extract the implied marginal mixture on any subset of features *h* for discriminative evaluation of clusters based on *A*_*c*_(*h*). When *p* is small, we can evaluate all possible 2^*p*^ subsets of features. For moderate to large *p*, an exhaustive search is impractical. The two most employed model/feature selection schemes are stepwise forward and backward feature selections. Backward search starts with the full set of features and deletes one feature at a time until no more needs be further removed (e.g., some stopping criterion has been met). Forward selection operates in a reversed manner by sequentially adding features one by one. For interpretation purposes, we would like to obtain the smallest possible subset of discriminative features. We thus propose to use forward selection. We stop forward selection if the increase in *A*_*c*_(*h*) is below a chosen threshold and/or if *A*_*c*_(*h*) exceeds a pre-specified high value. In this paper, our stopping criterion is that the change in *A*_*c*_(*h*) is below 0.1, and the absolute difference between *A*_*c*_(*h*) and *A*_*c*_(1:*p*) drops below 0.01.

For selecting potential biomarkers associated with the outcome, we employ logistic regression with elastic net regularization as mentioned in **Model training and testing**. The regularization can be written as $R(\beta_{1:C})=\sum_{c=1}^{C}\left[\lambda_{1}\sum_{j=1}^{p}\left(\frac{1-\lambda_{2}}{2}\beta_{c,j}^{2}+\lambda_{2}|\beta_{c,j}|\right)\right]$, where *λ*_1_ and *λ*_2_ are the tuning parameters. In GeM-LR, there are *C* number of logistic regressions fitted to different regions of the data space. Without any prior knowledge of the clusters, we perform the regularization independently to each regression model and assume the same level of sparsity across all *C* clusters. It is worth noting that GeM-LR can be easily adapted to incorporate multi-task learning regularization to jointly estimate the parameters across clusters [33, 34].

### 2.2. Other methods for comparison

We evaluate GeM-LR by comparing it with several widely used methods listed below.

1. ***Logistic regression***: Logistic regression (LR) modeling is widely used for analyzing multivariate data with binary outcomes. LR is a parametric model that estimates the class posterior probability of a data point (positive/negative or 1/0). Normally, a data point is classified as positive (label 1) if the estimated probability of the positive class exceeds 50%; otherwise, it is classified as negative (label 0). Under LR, it is assumed that there is a linear relationship between the logit transform of the class probability and the feature vector **X**. For high-dimensional data, elastic net regularization is employed and is implemented using lassoglm function in Matlab.
2. ***Cluster-then-predict***: Cluster-then-predict (CP) is an analysis framework in which data points are first clustered, and then a separate prediction model is built for each cluster. Unlike GeM-LR, the clustering step is unsupervised, which means the outcome information is not used. Furthermore, each cluster-specific prediction model is estimated using only data points in that cluster, and thus the approaches lack a mechanism to “borrow information” among the clusters. In this paper, we use Kmeans to perform clustering and build LR models using elastic net regularization (which is the same in GeM-LR) for each cluster.
3. ***K-Nearest neighbor classification***: K-Nearest Neighbor (KNN) classifies a data point to the class most dominant among its *K* nearest neighbor data points based on a pre-chosen distance measure. The balance between overfitting and underfitting is achieved solely by adjusting *K*. A smaller value of *K* tends to yield an overfitted model. *K* is often chosen by cross-validation or recommended to be the square root of the sample size. In particular, we use the MATLAB function fitcknn with the built-in OptimizeHyperparameters option to find the optimal distance metric and the value of *K*.
4. ***Random forest***: Random forest (RF) is an ensemble classification method with decision trees as the baseline classifiers. Each decision tree is trained on a random sample (with replacement) of the original data. In addition, at the split of each node in a decision tree, only a random subset of features is used. One major advantage of RF, using voting on the decisions of many trees, is its resistance to overfitting. We use the RF function in MATLAB called TreeBagger. We obtain the optimal number of trees and the minimum number of leaf node observations through a grid search strategy.
5. ***Shallow neural network***: A shallow neural network (SNN) contains only one input layer that stores the values of the feature vector of the data, a few hidden layers that processes the input, and an output layer that takes the response of the hidden layer as input. Each layer typically contains multiple nodes. In our analysis, the number of nodes for the input layer is the number of features. For binary classification problems, the output layer consists of only one node corresponding to either 0 or 1. SNN is implemented in MATLAB by the function fitcnet with the build-in OptimizeHyperparameters option to find the optimal number of layers and the number of nodes for each hidden layer.

### 2.3. Model evaluation

We use *K*-fold cross-validation (CV) to evaluate model prediction performance. CV is a common technique to test the effectiveness of a model when the sample size is small. To perform *K*-fold CV, we randomly split the dataset into *K* folds (groups). Out of the *K* folds, one fold is used as a hold-out or test dataset, and the remaining (*K*−1) folds are pooled together as a training set. We fit our model and the competing models on the training set and evaluate their prediction performance using the test set. We use the area under the receiver operating characteristic curve (AUC) as a model performance metric. In our analyses, *K* = 5. AUC under cross-validation can be computed in two ways: (1) by combining the results from each fold into a single ROC curve and calculating the corresponding AUC, or (2) by calculating the AUC for each fold separately and then averaging the results. We opt for the second approach, as recommended in [35], and denote the resulting cross-validated AUC as CV AUC. For model interpretation, we train a separate model using the entire dataset (after standardization) and base our interpretation on that model. However, this model is not used for evaluating predictive performance.

For methods that have tuning parameters, such as GeM-LR, *K*-fold CV can also be used to select the optimal tuning parameters. Such a procedure is called nested CV, which contains an inner loop CV for tuning parameters that is performed in each training dataset of the outer CV loop. The outer CV is used for measuring the overall performance of the model. With small datasets, however, nested CV may not be able to pick the best tuning parameters because of the (large) fluctuations in the measured model performance [36]. Thus, we tune parameters in a non-nested fashion. We perform CV on the entire data to select the optimal tuning parameters, then we assess the selected model performance through another independent CV. Specific to GeM-LR, by default, we set *λ*_2_ = 0.8 and apply CV on the entire data to select the optimal *λ*_1_.

## 3. Results

To demonstrate GeM-LR’s effectiveness for analyzing vaccine studies, in terms of both achieving high prediction performance and providing model interpretation for gaining insights, we apply GeM-LR to three datasets: (1) A proof of concept analysis (Section 3.1 HVTN 505 data); (2) A dataset of small sample size (Section 3.2 VAST data); (3) A dataset of low heterogeneity in the relationship between the input and the outcome (Section 3.3 CHMI data). For all three datasets, we standardize all the features based on the statistics of training data and apply the same transformation/scaling to the test data in that fold. As mentioned in Section 2.1 **Determine the number of clusters**, we use CV to select the optimal number of clusters for GeM-LR. Once the best GeM-LR model is identified, it is applied to the full dataset (after standardization) for interpretation. We evaluate and compare GeM-LR with competing methods (listed in Section 2.3) using measures described in Section 2.2.

### 3.1. HVTN 505 data

HVTN 505 (NCT00865566) was a preventive vaccine efficacy trial testing a DNA prime and adenovirus 5 (Ad5) boost (DNA/rAd5) vaccine regimen in men or transgender women who have sex with men in the United States from 2009 to 2013. Even though the vaccine failed to provide overall protection compared to the placebo [37], several subsequent analyses have been conducted to investigate the immune responses elicited by the vaccine [37–40]. Interestingly, the association of antibody-dependent cellular phagocytosis (ADCP) with the risk of HIV-1 infection varied in strength depending on the values of plasma HIV-1 Env gp140–specific IgA response [40]. The same observation holds for the association of Fc*γ*RIIa binding with the risk of HIV-1 infection. Specifically, the association of ADCP/Fc*γ*RIIa with the risk of HIV-1 acquisition was significantly reduced for vaccine recipients with low-to-undetectable Env gp140–specific IgA responses than those with detectable Env gp140–specific IgA. This finding suggests the existence of heterogeneity among the vaccinees. There are at least two clusters among the vaccinees: those with low-to-undetectable Env gp140–specific IgA and those with detectable Env gp140–specific IgA. In addition, the association between ADCP/Fc*γ*RIIa and vaccinees’ HIV-1 infection status varies between the two clusters. This scenario is precisely what motivated our proposed method. However, Neidich and colleagues [40] used the cluster-then-predict (CP) approach, which first manually dichotomized the Env gp140–specific IgA into negative and positive groups. Then the inverse probability of sampling weighted logistic regression was independently performed within each Env gp140–specific IgA group while adjusting for potential confounding factors: participant age, race, BMI, and behavioral risk score.

To build upon the results obtained from immune correlates analysis [40], we raise a couple of subtle questions that cannot be easily addressed by intuition. First, can we further improve the prediction performance of the conventional cluster-then-predict approach? In the absence of extra evidence, an implicit practical standard for adopting a model depends largely on its prediction power. The justification for treating subgroups differently based on Env gp140–specific IgA will be strengthened if a more principled modeling approach, such as GeM-LR, can achieve higher predictive performance than the existing CP approach. Second, is the separation by Env gp140–specific IgA into positive and negative groups the best choice? In other words, is there a better variable to form the groups? Third, is it possible that separation into more than two groups will yield even better predictions? To answer these questions and thus gain more insights, GeM-LR can help us from a formal modeling perspective.

**Fig 2:**
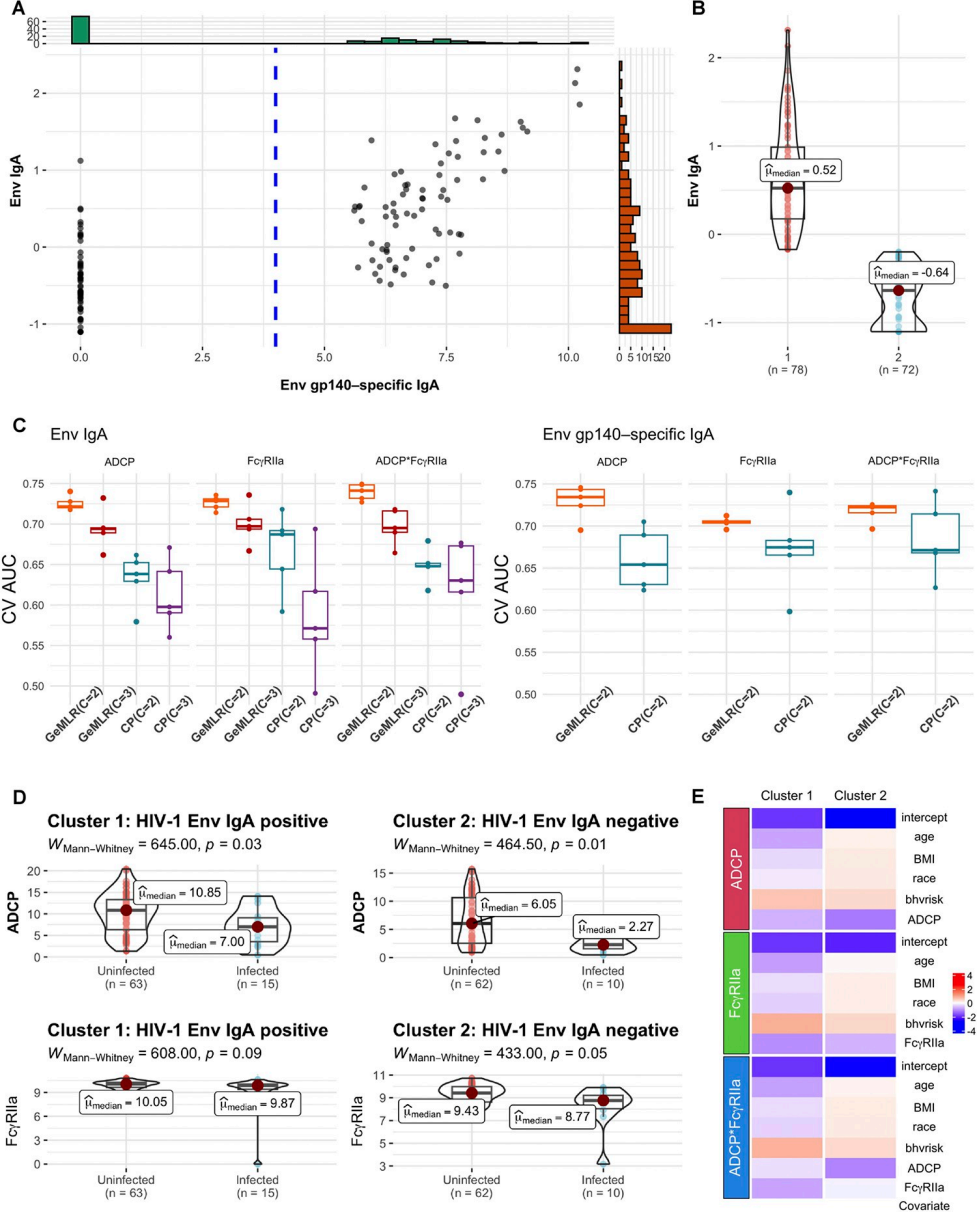
Visualizing GeM-LR results for HVTN 505 data. **A:** Scatter plot of Env IgA vs. Env gp-140-specific IgA. The cutoff value 4 for Env gp-140-specific IgA is shown in blue dotted line; **B:** Violin plot of HIV-1 Env IgA for the two clusters generated by GeM-LR; **C:** Box plots displaying the AUCs of the 5-fold CV for five repetitions, comparing GeM-LRs and the CP methods with *C* = 2:3: The left panel is for methods employing Env IgA as the clustering variable, whereas the right panel is for methods utilizing Env gp140-specific IgA for clustering. **D:** Violin plots of ADCP (top rows) and Fc*γ*RIIa (bottom rows) by the infected/uninfected outcome, with y-axis showing the values for ADCP and Fc*γ*RIIa, respectively, stratified by GeM-LR clustering result; p-values are computed by the nonparametric Mann–Whitney test comparing whether ADCP/Fc*γ*RIIa differs in the two clusters; **E:** Heatmaps for visualizing the regression coefficients in the three GeM-LRs.

**Experiment setup**: We apply GeM-LR to the HVTN 505 data, which was downloaded from https://atlas.scharp.org/cpas/project/HVTN%20Public%20Data/HVTN%20505/begin.view. Specific to this data, we do not impose elastic net regularization for GeM-LR, CP, and LR due to the relatively low number of features. The data consists of 150 vaccine recipients, of which 25 acquired HIV-1 infection. In the subsequent analysis, we let *Y* = 1 represent HIV-1 infection, and *Y* = 0 otherwise. Thus, we model the risk of HIV-1 infection. In Neidich et al. 2019 [40], the positivity criterion was based on a single IgA binding variable, Env gp140–specific IgA, and the cutoff was chosen to be 4. In addition, the data also have a mean-centered summary of a panel of eight IgA binding antibodies, denoted as Env IgA. Env IgA would be a more comprehensive representation of IgA binding than Env gp140–specific IgA. Fig 2A visualizes the two variables, with the y-axis displaying the mean-centered summary value for Env IgA and the x-axis showing the measured value for the single IgA binding variable, Env gp140–specific IgA. A mass of individuals has the variable Env gp140–specific IgA equal to 0, a value defined as the “low-to-undetectable” Env gp140–specific IgA binding, while the rest of the individuals have a spread of values above 5, a range defined as the “detectable” IgA binding. To follow the setting in Neidich et al. 2019 [40], we first use Env gp140–specific IgA for fitting GMM and use ADCP alone, Fc*γ*RIIa alone, or both ADCP and Fc*γ*RIIa as covariates, along with the four confounding factors, to fit cluster-wise logistic regression models. As a result, we have three GeM-LR models. We further set the number of mixture components *C* to 2 or 3. Our objective is to determine whether *C* = 2 is the optimal number of clusters through a model-based approach by comparing the mean AUC for *C* = 2:3. The model-fitting algorithm for GeM-LR indicates that the maximum number of clusters is 2, as there is an empty cluster when setting *C* = 3. We then perform a similar analysis by replacing Env gp140–specific IgA with Env IgA in the GMM. We perform both GeM-LR and CP with *C* = 2:3. We use the CP approach to mimic the prior study [40] and use it as a baseline method. Further, to account for the two-phase stratified sampling design in HVTN505, an inverse-probability oversampling is performed for each training data to correct for the sample selection bias [41]. The inverse probabilities are the inverse of the sampling probabilities determined by the two-phase sampling plan. For each testing data, the weighted AUC is computed accounting for the sampling probability. We repeat this inverse-probability oversampling based K-fold CV five times. In Fig 2C–2E, we distinguish the three GeM-LR models by the covariates used and denote them as “ADCP”, “Fc*γ*RIIa”, and “ADCP*Fc*γ*RIIa”.

**Model performance**: Fig 2B displays the clustering results estimated by GeM-LR when using Env IgA as the clustering variable. The y-axis shows the mean-centered summary value for Env IgA. The optimal number of clusters being 2 is suggested by Fig 2C. In the left panel of Fig 2C, we provide the (weighted) CV AUCs for five 5-fold CV repetitions for the three GeM-LRs and CPs at *C* = 2:3 when Env IgA is used as the variable for clustering. The predictive performance achieved by CP is worse than that by GeM-LR, with p-values of 0.002, 0.032, and 0.0007 for models using ’ADCP,’ ’FcγRIIa,’ and ’ADCP*FcγRIIa’ as covariates, respectively. This comparison was based on a paired t-test of CV AUCs between the 2-component GeM-LR and CP models. The 2-component GeM-LR achieves the highest overall AUC among five repetitions for all three GeM-LR models, an observation consistent with the past assumption of two groups of vaccinees. In addition, all three GeM-LR models yield the same cluster memberships. To ensure consistency, we also included AUC results comparing GeM-LR with all competing methods in S1 Fig. These results confirm that GeM-LR generally outperforms the other methods, although its predictive performance is comparable to LR. Both GeM-LR and CP results, in terms of (weighted) AUCs, when using Env gp-140 specific IgA binding as the clustering variable are recorded in the right panel of Fig 2E, where the regression coefficient values are color-coded from red (larger) to blue (smaller). Notably, the clusters estimated by GeM-LR align with the previously established positivity criterion, as Env gp140-specific IgA binding exhibits a clear two-cluster structure. It is worth noting that the prediction performance for clustering vaccinees using Env IgA is similar to when using Env gp140-specific IgA binding, confirming the validity of using Env gp140-specific IgA for clustering vaccinees. Furthermore, when employing Env gp140-specific IgA as a clustering variable, GeM-LR outperforms CP, although they yield the same clusters. One explanation for the superiority of GeM-LR is that GeM-LR can incorporate information from all data points when estimating cluster-specific LR models. The clusters estimated by GeM-LR when using Env IgA largely coincide with the prior finding that clusters 1 and 2 correspond respectively to the groups of lower and higher Env IgA, although the cutoff value between the two groups is different than from using Env gp140-specific IgA binding. Considering that Env IgA includes all available epitopes on the antigen, the result suggests that the IgA induction itself may also be a valuable factor for grouping the vaccinees.

**Insights by model interpretation**: Fig 2D and 2E display the model outputs when using Env IgA as a clustering variable. Fig 2D shows that the extent of association between ADCP and HIV-1 infection risk is stronger among vaccinees in cluster 2 than those in cluster 1. This finding is further confirmed by Fig 2E, which displays the regression coefficients for both clusters. Specifically, for the Env IgA negative group, the coefficients for ADCP, when it is the primary factor as well as when using both ADCP and Fc*γ*RIIa in the regression model, are larger in absolute value than those in the Env IgA positive group, implying that the association between ADCP and HIV-1 infection risk is stronger in cluster 2. Moreover, the difference between the two clusters reduces in terms of the association between Fc*γ*RIIa and the HIV-1 infection outcome, when Fc*γ*RIIa is the primary factor used in the logistic models. Fc*γ*RIIa shows some difference between the two clusters when using both ADCP and Fc*γ*RIIa in the regression model.

For this example, GeM-LR confirms prior findings from a relatively rigorous aspect of modeling. In addition, GeM-LR suggests that Env IgA, a more representative variable, can also be used for clustering since the best prediction performance is obtained by clustering Env IgA. In addition, GeM-LR reveals potential limitations of ad-hoc analysis and points to ways of enhancing existing analysis. This study also showcases that prior domain knowledge can be incorporated seamlessly into a statistical modeling approach via GeM-LR. More specifically, GeM-LR can serve as a tool to assess a conjecture or to leverage intuitions for improvement in outcome prediction. In this study, Env IgA is a continuous variable and does not exhibit any clear clustering patterns, nevertheless, GeM-LR is able to identify clusters helpful for predicting the outcome.

### 3.2. VAST data

*Salmonella enterica* serovar Typhi (S. Typhi) causes typhoid fever, which is a significant public health concern in many low- and middle-income countries. The subunit Vi polysaccharide (Vi-PS) vaccine is one of the most commonly used vaccines for typhoid fever globally. However, Vi-PS exhibited efficacy of around 69% in adults during the first year after vaccination, but its effectiveness declined over time and it is unsuitable for use in children under 2 years of age [42, 43]. For this, a Vi-tetanus toxoid (Vi-TT) conjugate vaccine has been developed, which is immunogenic and can be used from infancy. In a recent Vaccines against Salmonella Typhi (VAST) trial, Vi-TT was evaluated for vaccine efficacy in 72 healthy adult volunteers who were vaccinated with either Vi-PS or Vi-TT and then received the oral challenge with live S. Typhi bacteria in Oxford UK [44]. After the challenge, typhoid fever was diagnosed in 37% of Vi-PS vaccine recipients and 35% of Vi-TT vaccine recipients. The comparable efficacy of Vi-TT and Vi-PS vaccines despite an increased seroconversion rate and significantly higher geometric mean titers in Vi-TT vaccinees highlights the need for a better comprehension of the immune features linked with protection against typhoid fever.

**Fig 3:**
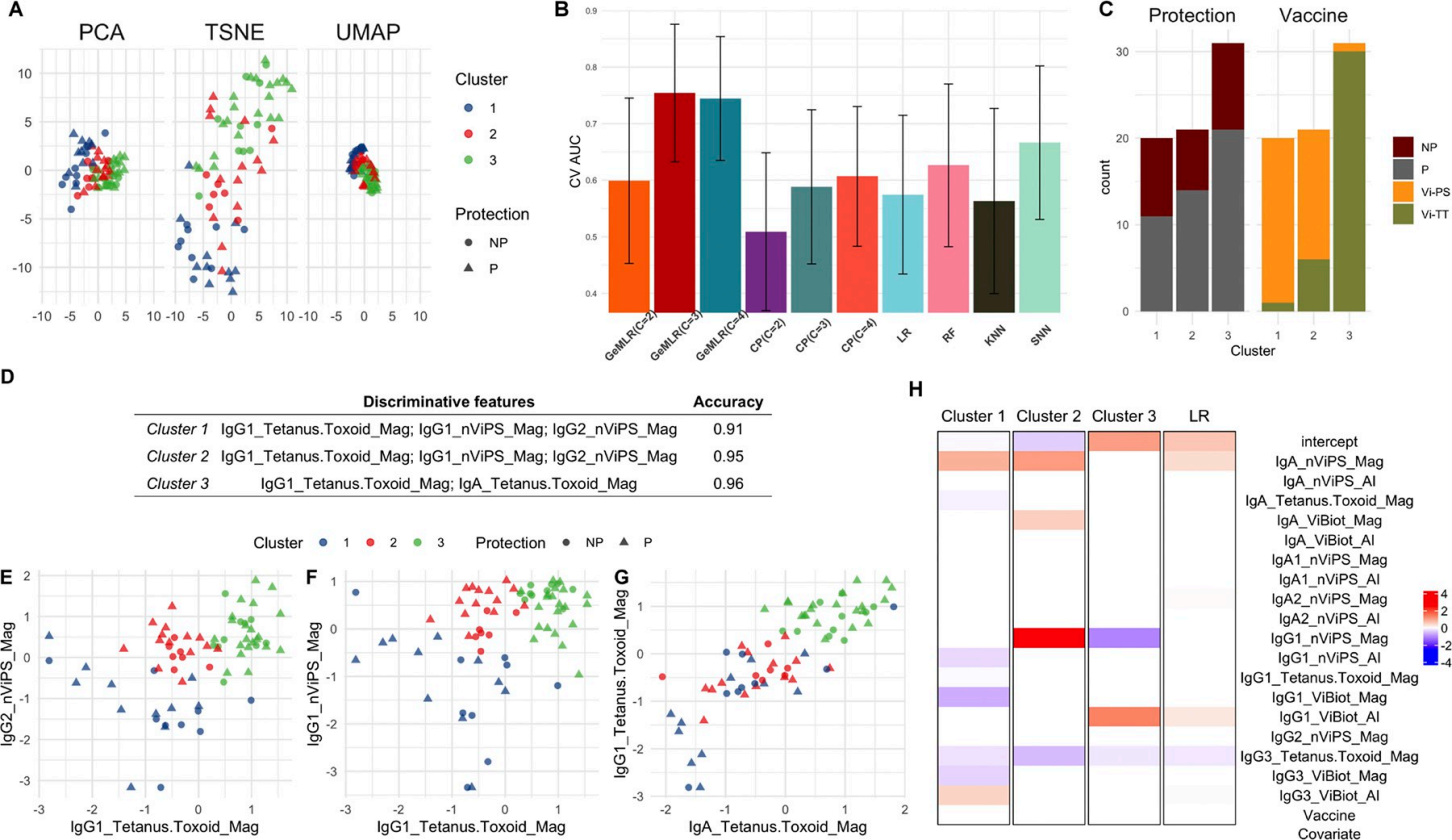
VAST data visualization and analysis results. **A:** Visualization by PCA, t-SNE, and UMAP with the protection status and cluster membership of individuals marked by different shapes and colors (NP: not protected; P: protected); **B:** Bar graphs showing the point (heights of the bars) and 95% confidence interval (black lines) estimates of CV AUCs by GeM-LR and the competing methods. **C:** Stacked bar chart highlighting the distribution of protection status and the two vaccines within each cluster; **D:** Identified discriminative features for all three clusters with the corresponding Accuracy *A*_*c*_(*h*); **E-G:** Scatter plots on the selected features with individuals color-coded by their cluster memberships. The x- and y-axes represent the normalized values of the corresponding variables. **H:** Heatmap for visualizing GeM-LR and LR regression coefficients; The regression coefficient values are color-coded from red (larger) to blue (smaller).

**Experiment setup**: We downloaded the VAST data [45] and utilized 18 immune features measured on the day of the oral challenge that were available for most participants, including IgA, IgA1, IgA2, IgG1, IgG2, and IgG3 magnitude (Mag) or avidity index (AI) of antibody binding to native Vi polysaccharide (nViPS) antigen, biotinylated Vi polysaccharide (ViBiot) antigen, and/or tetanus toxoid antigen, measured by a binding antibody multiplex assay (BAMA). We used the R package mice [46], employing the predictive mean matching function, to impute the missing values among the 18 immune features. We first project the 18 immunogenicity measurements into 2 (latent) dimensions for visualization using three different dimension reduction methods: principal component analysis (PCA), t-distributed stochastic neighbor embedding (t-SNE) [47] and uniform manifold approximation and projection (UMAP) [48]. Fig 3A depicts two-dimensional scatter plots of 72 individuals using three different visualization approaches. Shapes represent the individual protection status. All three visualizations provide similar information: there is no clear separation in the data.

We apply GeM-LR to the data with mixture components *C* ranging from 2 to 4. We let *Y* = 1 represent typhoid fever absence (protected) and *Y* = 0 being diagnosed with typhoid fever (not protected). Since this dataset is of a very small sample size with relatively high-dimensional features, we apply GeM-LR with covariance shrinkage for *C* = 2:4. We set the shrinkage level at 0.9. We also run GeM-LR, where GMM is modeled by a lower-dimensional feature. In this analysis, we select the top 5 highly variable features (measured by variance) for fitting GMM while using the entire 18 features and one binary indicator, “Vaccine”, to generate the logistic regression model. We set Vaccine = 1 to indicate Vi-PS vaccine recipient and Vaccine = 0 to indicate Vi-TT.

**Model performance**: We report each model’s prediction performance in terms of their CV AUC and the corresponding 95% confidence interval obtained by the cvAUC R package [49] using the non-nested CV procedure. The prediction performance is plotted in Fig 3B. The 3-component GeM-LR, denoted as GeM-LR (*C* = 3), achieves the highest CV AUC. Thus, we set *C* = 3 as the number of components (clusters). The individual’s cluster membership is color-coded in Fig 3A. Meanwhile, in Fig 3C, we see that cluster 1 mainly contains Vi-PS recipients and cluster 3 contains mostly Vi-TT vaccinees. The majority of cluster 2 consists of individuals who received the Vi-PS vaccine.

We also experimented with five competing methods: CP with *C* = 2:4, LR with an elastic net penalty, RF, KNN, and SNN. Among all competing methods, CP is the only one that also allows a different set of features used for performing clustering versus for predicting the outcome. For a fair comparison with GeM-LR, we applied CP with clustering conducted on the top 5 highly variable features. The corresponding prediction performance is shown in Fig 3B. All the competing methods do not perform well on this dataset based on the estimated CV AUC, however, their respective 95% confidence intervals overlap with that of the 3-component GeM-LR, indicating that the differences are not statistically significant.

The result in Fig 3B demonstrates the effectiveness of GeM-LR even when the data size is small. The framework of GeM-LR allows the implementation of multiple strategies to handle high-dimensional data. Here, in particular, we have employed variable selection and covariance regularization for the embedded GMM in addition to elastic-net regularization penalty for the cluster-wise logistic regression models. Although we also applied variable selection for the relatively simple CP method, the improvement in predictive performance is less remarkable. The disparity in prediction for GeM-LR and CP suggests that predictive clustering in GeM-LR contributes more to performance enhancement than purely unsupervised clustering in CP.

**Insights by model interpretation**: To better understand and obtain a clear interpretation for the three clusters, we apply forward variable selection to identify immune features (among the 5 features modeled by GMM) that are most informative for the partition of the data into the clusters. The results are summarized and visualized in Fig 3D–3G. Specifically, both clusters 1 and 2 can be accurately identified using the same set of three features. Fig 3E–3F shows that individuals in cluster 2 (which contains a small proportion of Vi-TT recipients) all have moderate IgG1 Tetanus Toxoid and moderate-to-high IgG2 nViPS and IgG1 nViPS bindings. In contrast, cluster 1 (dominated by Vi-PS recipients) all have lower IgG1 Tetanus Toxoid, IgG2 and IgG1 nViPS bindings. Similarly, Fig 3G shows that cluster 3 (dominated by Vi-TT recipients) can be identified by individuals with moderate-to-high IgG1 Tetanus Toxoid and IgA Tetanus Toxoid binding, consistent with their received vaccine regimen containing tetanus toxoid. Although there is no clear cluster-wise separation shown by any of the unsupervised visualization plots in Fig 3A, the three clusters can be discerned accurately in low dimensions according to Fig 3E–3G. Furthermore, although CP also identifies clusters (in an unsupervised manner), the predictive performance achieved by CP is considerably lower than that by GeM-LR. These observations show the advantage of GeM-LR for finding meaningful clusters in comparison with unsupervised clustering or popular visualization methods.

The three logistic regression models in GeM-LR with *C* = 3 select different sets of predictive biomarkers, as shown in Fig 3H. IgA nViPS has relatively larger coefficients for both clusters 1 and 2, suggesting that a stronger IgA nViPS binding is associated with protection for individuals belonging to the two clusters, which consists mostly of Vi-PS recipients. In contrast, the LR model also suggests the association of IgA nViPS with protection but with a smaller magnitude of coefficient. By comparing with the GeM-LR output, we see that the association may largely come from the Vi-PS vaccinees. In addition, a higher level of IgG1 nViPS binding is associated with an increased chance of protection for individuals belonging to cluster 2 only since IgG1 nViPS has the largest coefficient for cluster 2. On the other hand, it is interesting to note that individuals in cluster 3 who have higher values of IgG1 nViPS binding are more likely to get typhoid fever. These cluster-wise differences in the effects of the variables on the predicted outcome suggest that there is heterogeneity among vaccinees receiving each vaccine.

### 3.3. RTS,S/AS01 CHMI Study

RTS,S/AS01 is a piloted pre-erythrocytic malaria vaccine by World Health Organization which has demonstrated efficacy up to 86.7% in controlled human malaria infection (CHMI) studies of healthy malaria-naïve adults [50]. Although promising, the RTS,S vaccine does have important limitations. Vaccine efficacy is modest and short-lived. In children aged 5–17 months efficacy was 55% over the first 12 months after the primary course of vaccination (3 doses). Given the rapid waning of protective immunity, a booster dose is recommended 18 months after completing the primary immunization. When a booster dose was given, the RTS,S vaccine conferred 36% vaccine efficacy against symptomatic malaria and 29% efficacy against severe malaria over 4 years in the phase 3 trial [51]. Thus, it is important to identify the immune correlates of protection to inform the development of more efficacious and durable vaccines against malaria.

**Experiment setup**: We combine datasets from three RTS,S/AS01 CHMI clinical trials: NCT01366534 (referred to as MAL-068), NCT01857869 (referred to as MAL-071) and NCT03162614 (referred to as MAL-092). These datasets are publicly available on Zenodo at https://zenodo.org/records/10144807. The combined dataset comprises a total of 186 individuals, of which 111 were protected from the challenge. The dataset contains nine immune feature measurements, including binding magnitude and avidity index (AI) measured by BAMA and area under the dissociation curve measured by a biolayer interferometry (BLI) avidity assay, measuring antibody binding and avidity to CSP, NANP6, and N Interface peptides. Additionally, we also include two dummy variables (*I*_1_ and *I*_2_) to code for the three independent studies. Specifically, MAL-068 is treated as the reference group, with *I*_1_ = 1 and *I*_2_ = 0 for individuals from MAL-071, and *I*_1_ = 0 and *I*_2_ = 1 for individuals from MAL-092. In summary, we have 11 features to predict the protection status. Given our interest in identifying individual immune response heterogeneity, in the GeM-LR model, we use the nine immune features to fit the GMM and allow the cluster-wise logistic regression models to use additionally the identity of the studies (coded by *I*_1_ and *I*_2_). We set *Y* = 1 for protected vaccinees and *Y* = 0 for non-protected vaccinees.

**Fig 4:**
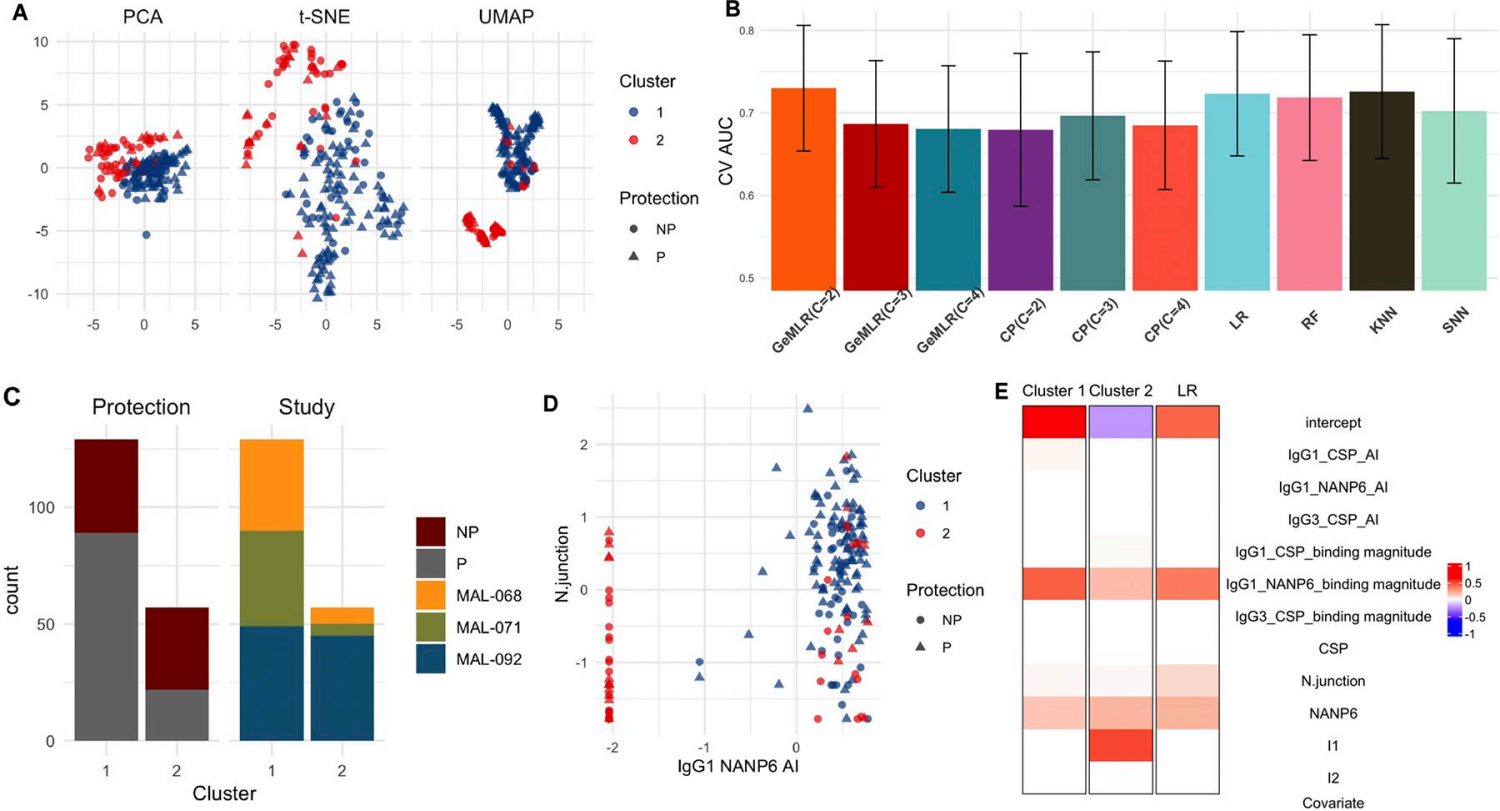
CHMI data. **A:** PCA, t-SNE, and UMAP visualizations with individual protection status and cluster memberships represented by shapes and colors (NP: not protected; P: protected); **B:** Bar graphs showing the point (heights of the bars) and 95% confidence interval (black lines) estimates of CV AUCs by GeM-LR and the competing methods. **C:** Stacked bar chart highlighting the distribution of protection status and the three studies within each cluster; **D:** Scatter plot on one of the identified discriminative features with individuals color-coded by their cluster memberships; The x- and y-axes represent the normalized values of the corresponding variables. **E:** Heatmap for visualizing GeM-LR and LR regression coefficients; The regression coefficient values are color-coded from red (larger) to blue (smaller).

**Model performance:** Similar to the VAST data analysis, we compute the CV AUC along with the corresponding 95% confidence interval using the cvAUC R package. Fig 4A presents data visualization based on the nine immune features, with triangles representing protected individuals, and circles representing non-protected individuals. Each of the three visualization results exhibits unique patterns, and both PCA and t-SNE analyses suggest that vaccinees are not clearly separated in the immune feature space. Moreover, the shapes are color-coded based on the cluster membership of individuals, as determined by GeM-LR with *C* = 2. Fig 4B provides the predictive performance for GeM-LR at *C* = 2:4 and all the competing methods. All methods exhibit similar performance in the sense of CV AUCs. The best GeM-LR model is that with *C* = 2.

**Insights by model interpretation**: Fig 4C shows that each of the three studies are represented in both clusters. Our proposed model selection analysis identifies three discriminative features. The scatter plot in Fig 4D shows the clustering results on one identified feature IgG1 NANP6 avidity index. In Fig 4E, we show side by side the sets of predictive biomarkers selected by the two cluster-wise LR models in GeM-LR (*C* = 2) along with the biomarkers selected by the LR fitted on the whole data. Both the cluster-wise LR models as well as the overall LR model share similar predictive biomarkers.

These results suggest that this dataset has little heterogeneity in the relation between input features and the outcome across different regions of the feature space. While GeM-LR achieves slightly better predictive performance at *C* = 2, the selected sets of biomarkers by the cluster-wise LR models are similar. Only *I*_1_ is selected for cluster 2 suggesting that individuals from cluster 2 being in the MAL-071 group is potentially associated with higher odds of being protected compared to the reference group, MAL-068. This example also highlights the subtlety of interpreting heterogeneity based on GeM-LR–it is inadequate to declare heterogeneity based solely on a selected *C*>1. One should also examine the similarity between the cluster-wise LR models. GeM-LR may favor *C*>1 for a small increase in predictive performance even though the LR models are similar. On the other hand, when *C* increases, each LR model is generally estimated using fewer data points which results in lower predictive performance. Therefore, if the data are truly homogeneous, by cross-validation, a small value of *C* will likely be chosen. In summary, this example shows that GeM-LR can maintain top performance even when the dataset exhibits little heterogeneity.

## 4. Discussion

We have proposed a novel framework of *Generative Mixture of Logistic Regression* (GeM-LR) for the analysis of small sample size vaccine studies. This new framework enables the identification of predictive clusters to account for data heterogeneity while allowing the use of cluster-wise linear models. By leveraging the strengths of both generative and discriminative modeling approaches, GeM-LR provides advantages including model interpretability and robust performance on small datasets. We have also developed procedures to identify discriminative features for cluster annotation and to find predictive biomarkers as potential correlates of protection (CoP). The ability to discover potential cluster-specific CoP can help us identify individuals in sub-populations who have responded to a specific vaccine.

For the HVTN505 dataset, our analysis by GeM-LR confirms previous findings that there are two groups of vaccinees based on the levels of Env gp140–specific IgA and the levels of associations between ADCP/FcγRIIa and the risk of HIV-1 acquisition are different among the two groups. GeM-LR, which performs soft-partition instead of hard-partition as in the CP method, increases the predictive performance significantly. In addition, GeM-LR identifies another variable for clustering, namely Env IgA, which has yielded a similar prediction performance. The optimal number of clusters based on Env IgA is also 2. However, different from Env gp140–specific IgA, Env IgA includes all available epitopes on the antigen and the vaccinees are clustered differently. This result suggests that the optimal grouping of the vaccinees may not be unique and IgA induction itself may also be an important variable. Further investigation may be needed to gain a deeper understanding of this finding.

Testing of a much smaller sample size from the VAST dataset demonstrated our GeM-LR methodology provided the highest prediction accuracy by clustering the data into three groups. The three clusters can be described based on the vaccine status of the participants. Cluster 1 contains a more homogeneous group of Vi-PS vaccinees, cluster 2 comprises a mixture of Vi-TT and Vi-PS vaccinees, while most participants in cluster 3 received the Vi-TT vaccine. This suggests that cluster 2 could contain immune responses common to both vaccine regimens or that individuals belonging to this cluster may be innately resistant to typhoid whereas clusters 1 and 3 are vaccine regimen specific responses. At the level of immune features, each cluster contains a different set of discriminative features and predictive biomarkers. These results not only confirm previous analyses that the two vaccines elicit different immune responses associated with protection [52, 53], but also suggest the possibility of identifying different sub-populations of individuals (even within each vaccine group) that most likely benefitted from typhoid vaccines. Specifically, IgA nViPS binding was identified previously to be significantly associated with protection in Vi-PS vaccinees, while it has no significant association with protection in Vi-TT vaccinees. On the other hand, higher values of anti-Vi IgG1 (IgG1ViBiot) avidity were observed to be associated with protection in Vi-TT vaccinees. Our GeM-LR analysis confirms the above findings. Our analysis further identifies IgG1 nViPS binding is most significantly associated with protection for individuals within cluster 2 while this marker decreases the chance of protection for individuals belonging to cluster 3 that are mostly Vi-TT vaccinees. In addition, nViPS-specific IgG2 binding was previously identified to be associated with protection in Vi-PS group. Our analysis suggests that IgG2 nViPS binding is an important variable for discriminating both clusters 1 and 2, which comprise mostly Vi-PS vaccinees. Interestingly, given the cluster membership, IgG2 nViPS binding is no longer a predictive biomarker associated with the outcome in all cluster-wise logistic regressions, indicating that IgG2 nViPS magnitude affects the outcome at the cluster level but not locally within each cluster. We recognize that caution should be taken when interpreting the predictive biomarkers because of the small sample size. Additional studies are needed to assess the robustness of the three sub-populations of data identified in the analysis and to validate the significantly different sets of predictive biomarkers in these sub-populations.

For the CHMI dataset, our analysis by GeM-LR confirms previous findings and provides additional insights. Prior studies using data from MAL-068 and MAL-071 have identified potential antibody measurement biomarkers associated with protection [54–56]. Using univariate and/or multivariate logistic regressions, IgG1 NANP6 binding magnitude was found to be the most predictive of the protection status [55]. Our analysis confirms this finding. In addition, we combine all three studies, including MAL-092, and the analysis suggests that there is no significant difference among studies in the association between antibody biomarkers and the protection status. This finding seems to suggest that IgG1 NANP6 binding magnitude may be a reproducible CoP within RTS,S CHMI trials in malaria-naïve adults. Confirmation of these results using GeM-LR based on data from trials of RTS,S or other malaria vaccines in malaria-endemic settings would be of interest to confirm this candidate CoP. On the other hand, our analysis reveals one difference. Previous studies show that NANP6 specific antibodies are more predictive of the outcome than N-junction specific antibodies [54, 57]. Our result aligns with this finding, showing that NANP6 has a larger effect than N-junction. More importantly, our analysis shows that N-junction is not significant for a subgroup of participants (cluster 2). Most participants belonging to cluster 2 have minimum values of IgG1 NANP6 avidity index. This observation suggests a potentially intricate relationship between the N-junction and the avidity of IgG1 antibodies to NANP6, warranting further investigation into the underlying mechanisms governing this phenomenon.

Building on the previous discussion of GeM-LR’s advantages, it is important to highlight its additional potential utility. For example, GeM-LR can identify predictive clusters, but we recognize that the interpretation of clusters, regardless of the computational method used, is inherently subjective and requires a thorough examination of biological relevance. One aspect of biological validation involves evaluating whether the clustered objects share common features that were not initially considered in the clustering process. This approach enhances the credibility of the clusters by linking them to meaningful biological patterns or processes. GeM-LR provides a practical framework for this process. Specifically, GeM-LR can use a subset of features for clustering (via the GMM model), while the LR model, using the full set of features, subsequently identifies shared characteristics within each cluster. This dual approach not only helps mitigate the curse of dimensionality commonly associated with clustering but also uncovers patterns that were not explicitly used during clustering, thereby expanding our understanding of the data. Clusters (i.e., subclasses) identified via GeM-LR can guide future experimental designs. For example, clusters with distinct characteristics in terms of vaccine response could inform targeted strategies for vaccine development, such as identifying which groups of individuals may require different vaccine formulations or dosing regimens to achieve optimal immunity. Additionally, understanding these subclasses could lead to the identification of biomarkers that predict vaccine efficacy, enabling more personalized and effective vaccination strategies. It is also worth noting that GeM-LR can serve as a tuning mechanism to refine clusters initially obtained using any advanced clustering method. This means GeM-LR can be seamlessly integrated with other clustering techniques to take advantage of recent developments. For example, when working with a large dataset, a deep neural network approach can be employed to evaluate variable importance and select the most relevant variables for clustering [58]. Additionally, multi-modal data can be utilized to generate initial clusters [59].

GeM-LR extends the capabilities of a linear logistic regression model to a non-linear classifier with adjustable complexity through the number of mixture components and the embedded GMM. This flexibility allows GeM-LR to handle potentially more diverse datasets. For instance, considering that vaccine responses can be influenced by various factors such as genetics, age, and environmental conditions, GeM-LR can be readily extended to incorporate multi-modal data analysis. For example, genetic information can be utilized for clustering, while other factors can be incorporated into the regression model within each cluster. Although GeM-LR maintains linear complexity with respect to the number of samples, high-dimensional feature spaces, such as those encountered with multi-modal data, present challenges, particularly in estimating covariance matrices during the EM algorithm. A common approach to address high dimensionality is to impose specific structural constraints on the covariance matrices, such as diagonal or block structures. We have implemented in the estimation of GeM-LR various options to constrain the structures of covariance matrices including diagonal matrices, a shared shape of covariance matrices across components, and/or a shared orientation of covariance matrices.

In summary, we have conducted experiments to compare our method with five other approaches, highlighting its advantages. Here, we restrict our attention to the problem of binary outcome classification with relatively homogenous populations of mostly healthy adults, and all examples demonstrate GeM-LR in a cross-validated context rather than its performance on new, external data. While exploring GeM-LR’s performance in external validation would be valuable, this is a noted limitation of the current paper. Our framework, however, is adaptable to a range of classification and regression problems in biomedical studies with different focuses. For instance, it is clinically important to examine vaccine-response heterogeneity from licensed vaccines in populations including individuals with various clinical factors, such as immunocompromise, dialysis, or cancer. Our method is promising for discovering such heterogeneities and may aid in identifying CoPs for designing vaccine candidates that target efficacy in priority populations more effectively.

## Supporting information

S1 Appendix This file includes a description of the formulation of GeM-LR, along with the expectation-maximization (EM) algorithm used for model fitting. (PDF)

S1 Fig Box plots displaying the AUCs of the 5-fold CV for five repetitions, comparing GeM-LRs with competing methods, for HVTN505 data. (DOCX)
