# Supplementary material for: GeM-LR: Discovering predictive biomarkers for small datasets in vaccine studies
Source: PLoS Comput Biol. 2024 Nov 14;20(11):e1012581. doi: 10.1371/journal.pcbi.1012581 (PMC11594404; doi:10.1371/journal.pcbi.1012581)
Supplement: S1 Appendix — (PDF) [file pcbi.1012581.s001.pdf]

Supplementary Information

1 GeM-LR and expectation-maximization (EM) algorithm for fitting the model

Let the sample points be $(\mathbf{x}_i, y_i)$, $i = 1, ..., n$, where $\mathbf{x}_i$ is the realization of the random feature vector $\mathbf{X}_i$, $\mathbf{X}_i \in \mathcal{R}^p$ and y_i is the realization of the class Y_i, $Y_i \in \{0, 1\}$. We describe the formulation of GeM-LR. For GeM-LR, we assume a latent component/cluster label Z, $Z \in \{1, 2, ..., C\}$, for a given sample point. An instance of Z is denoted by z. GeM-LR assumes that given $Z = c$, $c = 1, ..., C$, $\mathbf{X}$ follows a Gaussian distribution with mean $\boldsymbol{\mu}_c$ and covariance $\boldsymbol{\Sigma}_c$. In addition, GeM-LR assumes that given $Z = c$ and $\mathbf{X} = \mathbf{x}$, Y follows a logistic regression (LR) model with coefficients $\boldsymbol{\beta}_c$ (p-dimensional and excluding the intercept term) and $\boldsymbol{\beta}_{c,0}$ (intercept term). Consider a general sample point $(\mathbf{X}, Y)$,

$$P(Z = c) = \pi_c, c = 1, ..., C, \tag{1}$$

$$P(\mathbf{X}|Z = c) = N(\mathbf{X}|\boldsymbol{\mu}_c, \boldsymbol{\Sigma}_c), \tag{2}$$

$$P(Y = 1|\mathbf{X} = \mathbf{x}, Z = c) = \frac{\exp{(\boldsymbol{\beta}_c^T \mathbf{x} + \beta_{c,0})}}{1 + \exp{(\boldsymbol{\beta}_c^T \mathbf{x} + \beta_{c,0})}}. \tag{3}$$

Since we consider binary classification here, $P(Y = 0|\mathbf{X} = \mathbf{x}, Z = c) = 1 - P(Y = 1|\mathbf{X} = \mathbf{x}, Z = c)$. Based on the above Eqs. (1), (2) and (3), we can write down the joint probability for $\mathbf{X}$ and Y:

$$\begin{aligned} P(Y = 1|\mathbf{X}) &= \sum_{c=1}^{C} P(Z = c|\mathbf{X}) \cdot P(Y = 1|\mathbf{X}, Z = c) \\ &= \sum_{c=1}^{C} \frac{\pi_c N(\mathbf{X}|\boldsymbol{\mu}_c, \boldsymbol{\Sigma}_c)}{\sum_{c'=1}^{C} \pi_{c'} N(\mathbf{X}|\boldsymbol{\mu}_{c'}, \boldsymbol{\Sigma}_{c'})} \cdot \frac{\exp{(\boldsymbol{\beta}_c^T \mathbf{x} + \beta_{c,0})}}{1 + \exp{(\boldsymbol{\beta}_c^T \mathbf{x} + \beta_{c,0})}} . \end{aligned} \tag{4}$$

To estimate GeM-LR in Eq. (4), we use the EM algorithm with regularization where the latent state Z is regarded as missing data. Denote the parameters in GeM-LR collectively by $\theta = \{\pi_c, \boldsymbol{\mu}_c, \boldsymbol{\Sigma}_c, \boldsymbol{\beta}_c, \beta_{c,0}, c = 1, \ldots, C\}$, the mixture log likelihood of a point $(\mathbf{x}_i, Y_i)$, $i = 1, ..., n$, is given by

$$\begin{aligned} & l(\theta; \mathbf{x}_i, y_i) \\ = & \log \sum_{c=1}^{C} \pi_c N(\mathbf{x}_i \mid \boldsymbol{\mu}_c, \boldsymbol{\Sigma}_c) \cdot \Big[y_i P(Y_i = 1 \mid \mathbf{x}_i, z_i = c) + (1 - y_i) P(Y_i = 0 \mid \mathbf{x}_i, z_i = c) \Big], \end{aligned}$$

where we denote $P(Y_i = 1|\mathbf{x}_i, z_i = c) = p_{i,c}$ and $P(Y_i = 0|\mathbf{x}_i, z_i = c) = 1 - p_{i,c}$. The objective function (to be maximized), denoted by $\mathcal{L}$, is a penalized version of the sum of log likelihoods over all the points. Without loss of generality, let λ denote the (set of) tuning parameter(s) for the sparsity regularization on $\boldsymbol{\beta}_{1:C}$, denoted by $R(\boldsymbol{\beta}_{1:C})$. Since the penalty $R(\boldsymbol{\beta}_{1:C})$ is usually additive over the latent labels, we denote the penalty on $\boldsymbol{\beta}_c$ by $R(\boldsymbol{\beta}_c)$, and hence $R(\boldsymbol{\beta}_{1:C}) = \sum_{c=1}^{C} R(\boldsymbol{\beta}_c)$. The objective function based on λ is written as

$$\mathcal{L}(\lambda) = \sum_{i=1}^{n} l(\theta; \mathbf{x}_i, y_i) - \lambda R(\boldsymbol{\beta}_{1:C}). \tag{5}$$

For example, $\lambda R(\boldsymbol{\beta}_{1:C}) = \lambda \sum_{c=1}^{C} \sum_{j=1}^{p} |\beta_{c,j}|$ results in the $\mathcal{L}_1$ Lasso regularization, and $\lambda R(\boldsymbol{\beta}_{1:C}) = \sum_{c=1}^{C} \big[\lambda_1 \sum_{j=1}^{p} (\frac{1-\lambda_2}{2} \beta_{c,j}^2 + \lambda_2 |\beta_{c,j}|)\big]$ is the elastic net regularization. Elastic net is the same as Lasso when $\lambda_2 = 1$. As λ_2 shrinks toward 0, elastic net approaches ridge regression.

E step

Let $\theta^{(t)}$ denote the parameter values at the tth iteration. We first calculate the posterior probability distribution of z_i, $\gamma_{ci}^{(t)} \equiv P(z_i = c|y_i, \mathbf{x}_i, \theta^{(t)})$, $i = 1, ..., n$:

$$P(z_i = c|y_i, \mathbf{x}_i, \theta^{(t)}) \quad \propto \quad \pi_c^{(t)} N(\mathbf{x}_i|\boldsymbol{\mu}_c^{(t)}, \boldsymbol{\Sigma}_c^{(t)})[y_i p_{i,c}^{(t)} + (1 - y_i)(1 - p_{i,c}^{(t)})]\,, \;\; c = 1, ..., C.$$

To increase the impact of y_i on the choice of z_i, we transform the likelihood of Y by a shifted sigmoid function:

$$\frac{e^{\alpha(y_i p_{i,c}^{(t)} + (1-y_i)(1-p_{i,c}^{(t)}) - 0.5)}}{1 + e^{\alpha(y_i p_{i,c}^{(t)} + (1-y_i)(1-p_{i,c}^{(t)}) - 0.5)}}$$

where we set $\alpha = 1$ in our experiments. The complete log-likelihood of $(\mathbf{X}_i, Y_i, Z_i) = (\mathbf{x}_i, y_i, c)$ is

$$l(\theta; \mathbf{x}_i, y_i, c) = -\log(1 + \exp(\beta_c^T \mathbf{x}_i + \beta_{c,0})) + y_i(\beta_c^T \mathbf{x}_i + \beta_{c,0}) + \log N(\mathbf{x}_i|\boldsymbol{\mu}_c, \boldsymbol{\Sigma}_c) + \log(\pi_c)\,.$$

We then find the expectation of the complete data log-likelihood with respect to the posterior probability distributions of Z_i, $i = 1, ..., n$:

$$\begin{aligned} Q(\theta|\theta^{(t)}) &= \sum_{i=1}^{n} E_{Z_i|\mathbf{X}_i, Y_i, \theta^{(t)}} l(\theta; \mathbf{x}_i, y_i, Z_i) - \lambda R(\boldsymbol{\beta}_{1:C}) \\ &= \sum_{i=1}^{n} \sum_{c=1}^{C} \gamma_{ci}^{(t)} \cdot l(\theta; \mathbf{x}_i, y_i, c) - \lambda R(\boldsymbol{\beta}_{1:C})\,. \end{aligned}$$

M step

In the M step, we solve the new parameter $\theta^{(t+1)}$ by maximizing $Q(\theta|\theta^{(t)})$. For each latent label c, $c = 1, ..., C$, update the parameters as follows:

$$\begin{aligned} \boldsymbol{\mu}_c^{(t+1)} &= \frac{1}{n_c} \sum_{i=1}^{n} \gamma_{ci}^{(t)} \mathbf{x}_i\,, \;\; \text{where } n_c = \sum_{i=1}^{n} \gamma_{ci}^{(t)} \\ \boldsymbol{\Sigma}_c^{(t+1)} &= \frac{1}{n_c} \sum_{i=1}^{n} \gamma_{ci}^{(t)} (\mathbf{x}_i - \boldsymbol{\mu}_c^{(t+1)})(\mathbf{x}_i - \boldsymbol{\mu}_c^{(t+1)})^T \\ \pi_c^{(t+1)} &= \frac{n_c}{\sum_{c'=1}^{C} n_{c'}} \\ (\beta_{c,0}^{(t+1)}, \boldsymbol{\beta}_c^{(t+1)}) &= \underset{\beta_{c,0}, \boldsymbol{\beta}_c}{\operatorname{argmax}} \sum_{i=1}^{n} \gamma_{ci}^{(t)} \cdot [-\log(1 + \exp(\boldsymbol{\beta}_c^T \mathbf{x}_i + \beta_{c,0})) + y_i(\boldsymbol{\beta}_c^T \mathbf{x}_i + \beta_{c,0})] - \lambda R(\boldsymbol{\beta}_c)\,. \end{aligned}$$

Note that the optimization of $(\beta_{c,0}^{(t+1)}, \boldsymbol{\beta}_c^{(t+1)})$ is solved by fitting an elastic net model. Iterate E and M steps until the objective function $\mathcal{L}(\lambda)$ in Eq. 5 converges.

Initialization and Convergence Issues for EM

For a chosen C value, we apply Kmeans clustering algorithm on the feature matrix $\mathbb{X}$ to cluster the data points. We use the cluster means, covariance matrices and cluster proportions as the initial values of the parameters associated with GMM. To initialize the coefficients in the logistic regression models, we fit a model by elastic net using data points in every cluster. We run EM algorithm on 300 starting points/initializations, and we pick the seed that yields the best within-training classification accuracy according to AUC.
