# Supplementary material for: GeM-LR: Discovering predictive biomarkers for small datasets in vaccine studies
Source: PLoS Comput Biol. 2024 Nov 14;20(11):e1012581. doi: 10.1371/journal.pcbi.1012581 (PMC11594404; doi:10.1371/journal.pcbi.1012581)
Supplement: S1 Fig — (DOCX) [file pcbi.1012581.s002.docx]

**
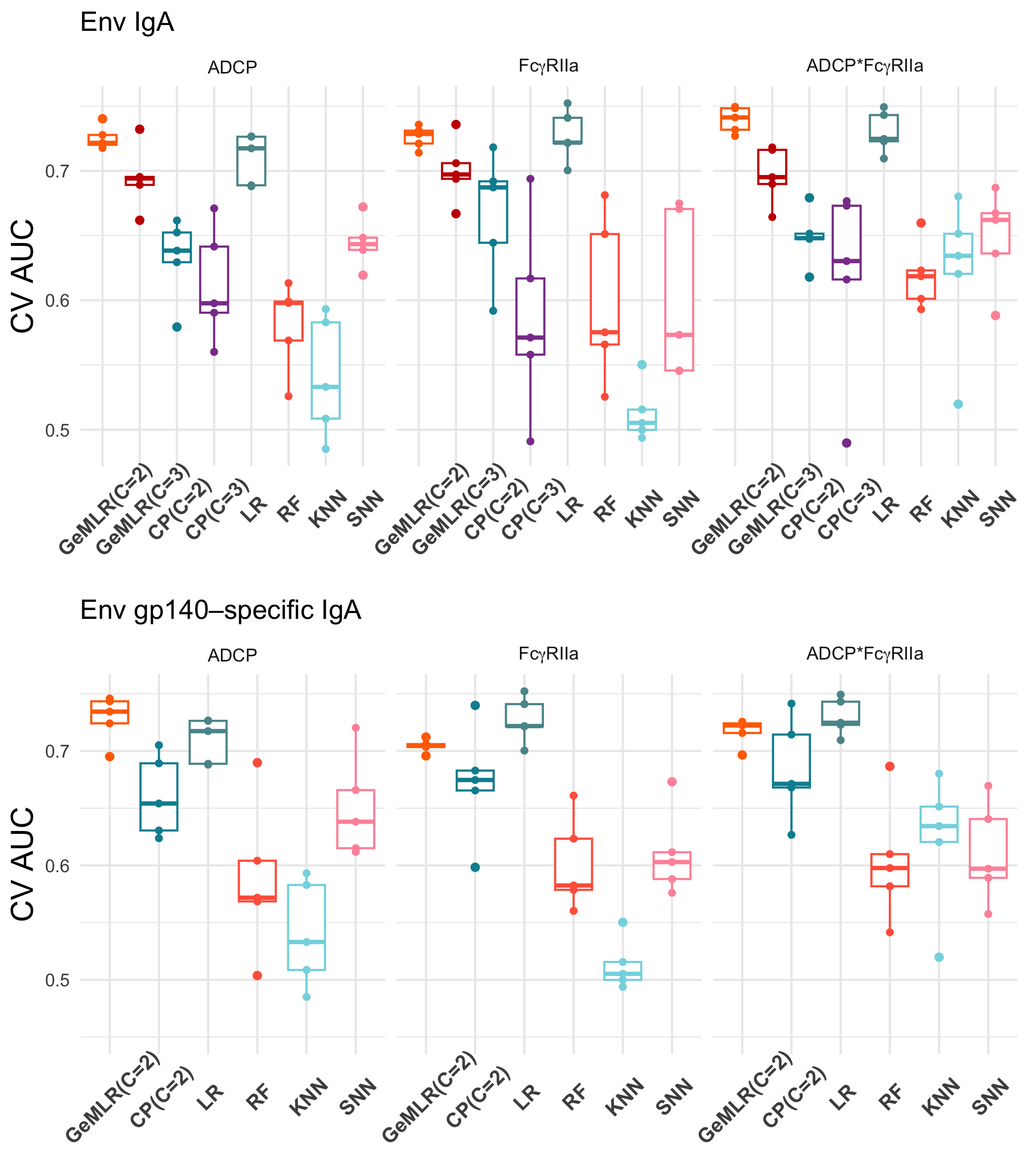
**

**S1 Fig. Box plots displaying the AUCs of the 5-fold CV for five repetitions, comparing GeM-LRs with competing methods, for HVTN505 data.**

The top panel displays results for methods using Env IgA as the clustering variable, while the bottom panel shows results for methods utilizing Env gp140-specific IgA.
